# Characterization and prediction of the clinical result with a specific model of mini-scleral contact lens in corneas with keratoconus

**DOI:** 10.1186/s40662-022-00310-5

**Published:** 2022-10-06

**Authors:** Abdelkader Sidi Mohamed Hamida, García-Barchín Marta, Ruiz-Fortes Pedro, David P. Piñero

**Affiliations:** 1 Department of Ophthalmology, Torrecárdenas Hospital Complex, 04009 Almería, Spain; 2 Group of Optics and Visual Perception, Department of Optics, Pharmacology, and Anatomy, University of Alicante, Crta San Vicente del Raspeig s/n 03016, San Vicente del Raspeig, 03690 Alicante, Spain; 3 Department of Ophthalmology, Vithas Medimar International Hospital, 03016 Alicante, Spain

**Keywords:** Keratoconus, Corneal ectasia, Scleral contact lens, Miniscleral contact lens, Aberrometry, Visual acuity

## Abstract

**Background:**

To investigate which factors are correlated with the visual improvement achieved with a specific model of scleral contact lens (SCL) in keratoconus (KC) eyes and to define a model to predict such improvement according to the pre-fitting data. In addition, the changes occurred with the fitting of a specific model of SCL during a period of 3 months in corneas with KC have been investigated.

**Methods:**

Longitudinal retrospective study including 30 eyes of 18 patients (age, 14–65 years) with KC fitted with the SCL ICD16.50 (Paragon Vision Sciences). Visual, refractive, corneal tomographic and ocular aberrometric changes were evaluated during a 3-month follow-up. Likewise, the characterization of the post-lens meniscus was performed by optical coherence tomography (OCT) with the measurement of central, nasal and temporal vaults.

**Results:**

The visual acuity increased significantly from a mean pre-fitting value with spectacles of 0.23 ± 0.07 logarithm of minimal angle of resolution (logMAR) to a mean value of 0.10 ± 0.04 logMAR after 1 month of SCL wear (*P* < 0.001). An improvement of 1 or more lines of visual acuity with the SCL occurred in 62.1% of the eyes. A significant decrease in central, nasal, and temporal vault was observed after 1 month of SCL wear (*P* ≤ 0.046). Likewise, there was a significant difference between nasal and temporal vaults during the first month of SCL use (*P* = 0.008). Furthermore, a significant reduction of ocular high order (*P* = 0.028) and primary coma root mean square (*P* = 0.018) was found with the SCL. A predicting linear equation of the change in visual acuity achievable with the SCL was obtained (*P* < 0.001, R^2^ = 0.878) considering the pre-fitting spectacle corrected distance visual acuity, and the power and sagittal lens of SCL.

**Conclusions:**

The scleral contact lens evaluated provides an efficacious visual rehabilitation in KC due to the improvement of visual acuity and the correction of low and high-order ocular aberrations. This visual acuity improvement can be predicted from some pre-fitting variables.

## Background

Scleral contact lens (SCL) are currently reaching a great impact due to their indication in irregular corneas such as keratoconus (KC), pellucid marginal degeneration, keratoglobus, post-keratoplasty or postsurgical ectasia, and in patients with severe ocular surface disease, such as extreme dry eye syndrome, Sjögren syndrome, Stevens-Johnson syndrome, scarring ocular pemphigoid, neurotrophic corneal disease, and atopic keratoconjunctivitis [1–6]. SCL have increased in popularity in the last decade, and it was estimated that 70,000 individuals in the United States wore a SCL in 2016 [7, 8]. As SCL is a large-diameter gas-permeable device specially designed to rest on the sclera and vault over the entire corneal surface, not bearing on the corneal structure and consequently respecting it, it is an ideal option for visual rehabilitation in KC [9]. Indeed, KC is the main indication of the use of these lenses [7, 8, 10–12]. Koppen et al. [13] reported that SCLs mitigated the need for corneal transplant in 80% of patients with severe KC.

Several studies have demonstrated that a successful visual rehabilitation can be achieved with SCLs in KC [6, 7, 14–16], even in cases with previous surgical treatments, such as intracorneal ring segments [17, 18]. However, the visual improvement achieved with SCLs can vary significantly between individuals [6, 7, 14–18]. Several factors may account for this, such as the selection of the vault [19, 20]. Otchere et al. [19] concluded in a clinical study that a SCL fitted adding 375 µm to the corneal sagittal height measured by optical coherence tomography (OCT) gave the best combination of visual acuity and comfort ratings. However, Sonsino and Mathe [20] did not find a correlation between the vault magnitude and logMAR visual acuity, including in their study patients fitted with SCLs with vaults up to 600 µm and as low as 220 µm. Therefore, other factors are involved in the level of visual recovery provided by SCLs in KC. The current study aimed to investigate which factors are correlated with the visual improvement achieved with a specific model of SCL and to define a model to predict such improvement according to the pre-fitting data.

## Methods

### Study population

The study was designed as a longitudinal retrospective study of the subjects with KC examined at the Advanced Clinical Optometry Unit of the Department of Ophthalmology of the Vithas Medimar International Hospital (Alicante, Spain) and fitted with a specific model of SCL (ICD16.50, Paragon Vision Sciences, distributed in Spain by Lenticon, Madrid, Spain). The research was carried out in accordance with the principles of the Declaration of Helsinki, obtaining written consent from all patients for the use of their data in this retrospective analysis. The study was approved by the ethics committee for medical research of the Health Department of Alicante (General Hospital, Alicante, Spain) (CEIm 2020-048, ISABIAL 200045).

The inclusion criteria for the study were patients with KC in any degree of progression, registered in our database, and fitted with the ICD16.50 SCL. No patients in our study presented with corneal scars at the beginning of the SCL fitting. Patients with or without previous corneal collagen cross-linking (CXL) surgery or implantation of intracorneal ring segments were included. The exclusion criteria were keratoplasty, post-refractive surgery corneal ectasia, contact lens (CL) fitting with another design of SCL or a follow-up of the CL wear of less than 3 months. Only one eye per patient was randomly included in patients with bilateral KC to avoid the potential bias associated to the inclusion of data of both eyes of the same patient that are normally correlated. In unilateral KC, the eye affected was included.

### Clinical examinations

**Fig. 1:**
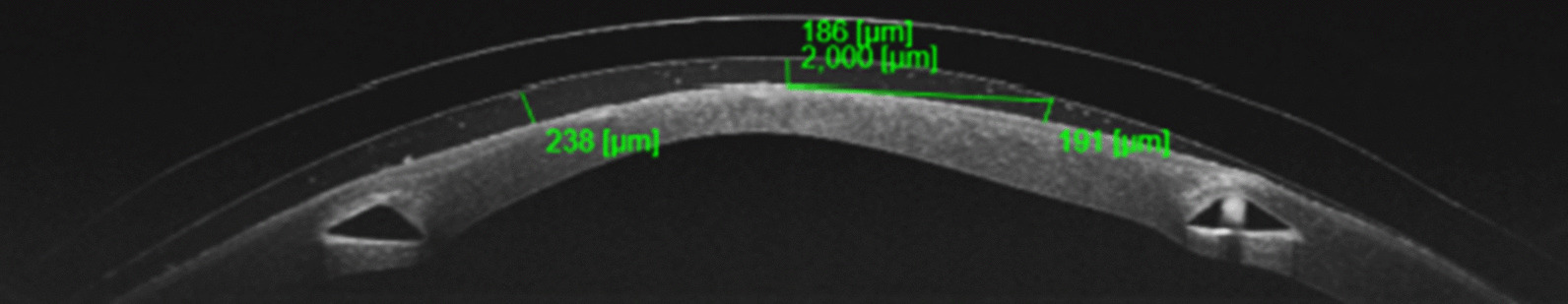
Example of characterization of the post-lens meniscus by measuring the vault centrally and at 2 mm nasally and temporally

All patients had undergone a complete pre-fitting examination including measurement of uncorrected (UDVA) and corrected distance visual acuity (CDVA), manifest refraction, slit lamp biomicroscopy, corneal topography and pachymetry with the Sirius tomographer (CSO, Firenze, Italy), and ocular aberrometry with the i-Trace system (Tracey Technologies Corp., Houston, TX, USA). After the selection of the first trial lens according to the manufacturer guidelines and subsequent trials if needed, the visual acuity with the scleral lens (SLVA) was measured as well as the over-refraction and the central vault using OCT (DRI OCT Triton, Topcon). Lenses were fitted by varying the sagittal height based on corneal clearance. Each lens cleared the steepest point of the cornea and provided the most uniform thickness profile of fluid accumulation between the lens and cornea. Corneal clearance was evaluated using a 45º degree oblique slit beam, and the desired clearance was 250 to 350 μm based on the central lens thickness. The landing zones of the individual lenses were determined by observing the vascular compression patterns of the bulbar conjunctiva with the goal of minimizing lens induced compression of conjunctival blood vessels and enabling unobstructed blood circulation. Once the SCL with the appropriate optical power and the required adjustments of the periphery were completed, measurements of SLVA, ocular aberrometry and post-lens meniscus was performed. The post-lens meniscus was characterized via the measurement of the central vault and the vault at 2 mm nasally and temporally from the center (Fig. 1). These measurements were performed along the horizontal meridian considering the pupillary center as a central reference. These measurements were taken with the OCT caliper option by a single researcher and supervised by a professional with experience in the CL field. All measurements were performed in the afternoon-evening after 6 h of CL wear during the day.

After this first assessment with the final CL prescribed, additional examinations were performed after 1 and 3 months of CL wearing. In these two post-fitting visits, SLVA measurement, slit lamp biomicroscopy, OCT-based post-lens meniscus characterization, and corneal tomography without the CL were performed.

The following parameters were collected and recorded from the Sirius tomography system: anterior (KMa) and posterior mean keratometry (KMp) for the central 3-mm area, anterior (ASTa) and posterior corneal astigmatism (ASTp) for the central 3-mm area, anterior (Qa) and posterior corneal asphericity (Qp) for the central 8-mm area, central (CCT) and minimal corneal thickness (MCT), and anterior and posterior corneal surface aberrations (6-mm pupil), including high-order aberration (HOA), primary coma, and residual aberration (all HOAs except primary coma and spherical aberration) root mean square (RMS) as well as the Zernike term corresponding to the primary spherical aberration (SA) (Zernike polynomials).

### Scleral contact lens

The ICD16.50 lens is a miniscleral contact lens manufactured by KATT Design Group with the Paragon HDS 100 (Paflufocon D) material that has a Dk of 100 Fatt units, forward wetting and backward wetting angles of 42º and 70º, respectively, specific gravity of 1.10, hardness (Shore D) of 79 and water content of < 1% [21]. The lens has a diameter of 16.50 mm, thickness of 0.29 mm and is available in powers from − 40 to + 30 D in 0.25-D steps, and in sagittal heights from 3900 to 5600 μm [16]. For a customized optimal adjustment, the lens has 4 differentiated zones that can be modified: central clearance zone (CCZ), peripheral corneal clearance zone (PCCZ), limbal clearance zone (LCZ) and scleral landing zone (SLZ) [16]. This SCL has been designed to be fitted mainly in irregular corneas [6, 16, 22–25]. The fitting was performed following the manufacturer guidelines: selection of the initial diagnostic lens according to the fitting table, insertion of the CL, evaluation of CCZ, assessment of central clearance, slit lamp examination of the SCL after 60 min of wearing and finally measurement of the over-refraction (ORx) to determine the final power of the CL [16].

### Statistical analysis

All data were analyzed by IBM-SPSS version 24.0 statistical software (SPSS Inc., Chicago, IL). Frequency distribution tables were used to describe the data. To summarize the information of the variables, average measures (mean and median) and dispersion measures [variance, standard deviation (SD), and range] were used. The normality of the data distributions was checked using the Kolmogorov-Smirnov test. When the variables followed a normal distribution, the Student’s t-test for paired samples was used to compare the data between consecutive visits. In the case of non-normalized distributions, the Wilcoxon rank test was used to compare parameters between consecutive visits. Likewise, the correlation between different clinical variables was analyzed, using the Pearson or Spearman correlation coefficients depending on whether the analyzed data distributions followed normality or not.

Finally, a multiple linear regression analysis was carried out to check whether the achieved change in visual acuity could be estimated based on different pre-fitting clinical variables. This analysis was performed using the backward step technique. Once the predictive formula was obtained, it was verified whether it met the necessary conditions for its use: homoscedasticity (normal distribution of non-standardized residuals), absence of multicollinearity (tolerance and inflation variance factor), absence of outliers (Cook's distance) and absence of correlation between errors (Durbin-Watson test).

## Results

A total of 30 eyes from 15 patients (10 men and 5 women) with KC were included. A total of 17 (56.7%) right eyes and 13 (43.3%) left eyes were considered for the analysis. The age of patients ranged from 14 to 65 years old, with a mean value of 34.5 years (SD: 6.7 years). Of the 30 eyes studied, 15 (50%) had KC without previous treatment, 10 (33.3%) had been treated previously with CXL, 5 (16.7%) had been previously treated with implantation of intracorneal ring segments. According to the Amsler-Krumeich grading system, the distribution of the severity of the KC was as follows: grade I (13 eyes, 43.3%), grade II (15 eyes, 50.0%), grade III (1 eye, 3.3%), and grade IV (1 eye, 3.3%). The initial sphero-cylindrical refraction and CDVA data are shown in Table 1.

**Table 1:** Pre-fitting visual and refractive data

Variable	Mean (SD)	Median (range)
Sphere (D)	− 2.64 (1.42)	− 1.37 (− 12.00 to 3.00)
Cylinder (D)	− 3.01 (0.65)	− 3.00 (− 6.50 to 0.00)
CDVA (logMAR)	0.23 (0.07)	0.22 (0.00 to 0.70)

*CDVA* = corrected distance visual acuity; *D* = diopters; *logMAR* = logarithm of minimal angle of resolution; *SD* = standard deviation

**Fig. 2:**
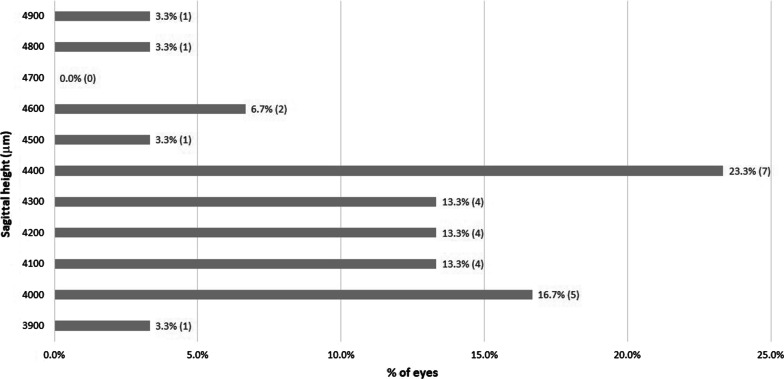
Distribution of the sagittal heights of the scleral contact lenses fitted in the study

The mean power of the fitted lenses was − 3.04 D (SD: 1.78 D; median: − 2.38 D; range: − 15.50 to 4.25 D). The SCLs used were mostly spherical and only 5 (16.7%) eyes required peripheral toricity. Regarding the design of the lenses, the sagittal height used ranged from 3,900 to 4,900 μm. The distribution of the sagittal heights used in the study is shown in Fig. 2. Adjustments of SLZ were needed in a total of 12 eyes (40.0%).

### Visual acuity changes

**Fig. 3:**
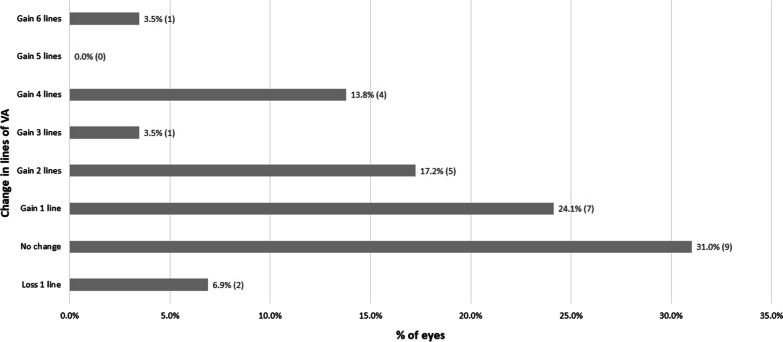
Distribution of VA changes achieved with the scleral lenses fitted in the study after 1 month of SCL use. VA, visual acuity

The visual acuity (VA) increased significantly from a mean pre-fitting value of 0.23 logMAR (SD: 0.07 logMAR) to a mean value of 0.10 logMAR (SD: 0.04 logMAR) after 1 month of CL wear (*P* < 0.001) (Table 2). The mean VA difference was − 0.13 logMAR (SD: 0.07 logMAR) after 1 month of CL wear and the median was − 0.10 logMAR, indicating a mean increase in VA with the CL of around 1 line. However, the increase in VA ranged from − 0.60 to 0.10 logMAR, and patients could gain in some cases 6 lines of VA (Fig. 3). An improvement of 1 or more lines of VA (logMAR) occurred in 62.1% of the eyes, whereas only one case lost 1 line of VA. Between the first and third month of CL wear, no significant changes were detected in visual and refractive outcomes (*P* ≥ 0.180; Table 2).

**Table 2:** Visual acuity and over-refraction data with the final scleral contact lens fitted during the follow-up

Variable	1 month follow-up			3 months follow-up		
	Mean (SD)	Median (Range)	*P* value	Mean (SD)	Median (range)	*P* value
SLVA	0.10 (0.04)	0.10 (− 0.08 to 0.30)	< 0.001	0.10 (0.06)	0.10 (− 0.08 to 0.40)	0.219
Sphere ORx (D)	0.22 (0.16)	0.00 (− 0.50 to 1.50)	< 0.001	0.18 (0.21)	0.00 (− 0.50 to 1.00)	0.357
Cylinder ORx (D)	− 0.05 (0.08)	0.00 (− 1.00 to 0.00)	< 0.001	− 0.14 (0.20)	0.00 (− 1.25 to 0.00)	0.180
SLVA with ORx (logMAR)	0.09 (0.04)	0.10 (− 0.08 to 0.30)	–	0.07 (0.05)	0.04 (− 0.08 to 0.30)	0.753

*SLVA* = scleral lens corrected distance visual acuity; *ORx* = Over-refraction; *logMAR* = logarithm of minimal angle of resolution; *SD* = standard deviation; *D* = diopters

### Post-lens meniscus characterization

Measurement of the post-lens meniscus by OCT showed a statistically significant decrease in central, nasal, and temporal vault after 1 month of CL wear (*P* ≤ 0.046; Table 3). Mean decrease in central vault after 1 month of CL wear was − 84.09 μm (SD: 82.45). However, between the first and third month of CL wear, there was a tendency of these vault measures to increase but were lacking statistical significance (*P* ≥ 0.131; Table 3).

**Table 3:** Measurements of the central, nasal and temporal vault of the scleral contact lens over the cornea in the initial fitting and after 1 and 3 months of contact lens wear

Variable	First trial		1 month follow-up				3 months follow-up			
	Mean (SD)	Median (range)	Mean (SD)	Median (range)	Change compared to first trial	*P* value	Mean (SD)	Median (range)	Change compared to first trial	*P* value
Central vault (μm)	294.46 (33.13)	267.00 (167 to 543)	200.09 (36.85)	207.00 (111.0 to 307)	− 84.09 (82.45)	0.046	228.0 (60.04)	207.50 (122 to 361)	+ 19.55 (30.22)	0.174
Nasal vault (μm)	278.50 (51.00)	276.50 (176 to 441)	194.45 (27.32)	198.0 (126 to 240)	− 80.64 (63.89)	0.018	227.50 (42.47)	228.0 (137 to 328)	+ 26.22 (35.98)	0.131
Temporal vault (μm)	344.42 (58.28)	306.50 (267 to 587)	257.36 (48.32)	245.0 (150.0 to 394.0)	− 80.36 (87.21)	0.021	269.70 (67.34)	249.5 (143 to 417)	+ 3.89 (38.05)	0.820

*SD* = standard deviation

As shown in Table 3, the vault values showed a variation along the horizontal axis, with lower values for the nasal area and higher values for the temporal area in all visits. The difference between nasal and temporal vault was statistically significant in the first trial and after 1 month of CL wear, with mean values of 65.92 µm (SD: 43.35 µm; median: 84.50 µm; range: 58 to 152 µm; *P* = 0.01) and 62.91 µm (SD: 42.60 µm; median: 55.50 µm; range: 24 to 167 µm; *P* = 0.008), respectively. After 3 months of CL wear, this difference between nasal and temporal vaults decreased, but did not reach statistical significance (mean: 42.40 µm; SD: 47.30 µm; median: 40.00 µm; range: 55 to 172 µm; *P* = 0.074).

### Corneal tomographic and aberrometric changes

No statistically significant changes were detected in the tomographic data evaluated (*P* ≥ 0.276); Table 4). There was a tendency for asphericity towards more negative values after 3 months of CL wear, but it did not reach statistical significance (*P* ≥ 0.635). Concerning aberrations, HOAs trended higher after 3 months of CL wear but did not reach statistical significance (*P* ≥ 0.110; Table 5).

**Table 4:** Tomographic data before fitting and after 3 months of contact lens wear

Variable	Pre-fitting		After 3 months of CL wear		
	Mean (SD)	Median (range)	Mean (SD)	Median (range)	*P* value
Km_ant_ (D)	48.91 (1.64)	48.80 (42.03 to 63.24)	50.28 (5.08)	48.87 (42.86 to 64.47)	0.594
Ast_ant_ (D)	4.60 (0.98)	4.11 (1.12 to 12.55)	6.55 (3.44)	5.20 (0.87 to 13.69)	0.513
Q_ant_	− 0.93 (0.22)	− 0.92 (− 2.28 to 0.61)	− 1.23 (0.54)	− 1.06 (− 2.56 to 0.43)	0.813
Km_post_ (D)	− 7.54 (0.39)	− 7.54 (− 11.32 to − 6.17)	− 7.83 (1.17)	− 7.89 (− 11.32 to − 6.22)	0.575
Ast_post_ (D)	0.93 (0.17)	1.03 (0.09 to 1.66)	1.07 (0.37)	1.14 (0.40 to 1.90)	0.735
Q_post_	− 1.15 (0.25)	− 0.98 (− 3.18 to − 0.44)	− 1.41 (0.61)	− 1.21 (− 3.04 to − 0.49)	0.635
MCT(μm)	442.79 (24.98)	455.00 (313 to 554)	421.89 (48.52)	432.00 (322 to 551)	0.276
CCT (μm)	485.38 (26.05)	484.00 (340 to 558)	457.55 (42.27)	481.00 (342 to 556)	0.343

*SD* = standard deviation; *D* = diopters; *Q*_*ant*_ = asphericity of the anterior corneal surface; *Q*_*post*_ = asphericity of the posterior corneal surface; *Km*_*ant*_ = anterior mean keratometry; *Km*_*post*_ = posterior mean keratometry; *Ast*_*ant*_ = anterior astigmatism; *Ast*_*post*_ = posterior astigmatism; *MCT* = minimal corneal thickness; *CCT* = central corneal thickness

**Table 5:** Corneal aberrometric based on Zernike polynomials data before-fitting and after 3 months of contact lens wear

Variable (6-mm pupil)	Pre-fitting		After 3 months of CL wear		
	Mean (SD)	Median (range)	Mean (SD)	Median (range)	*P* value
Anterior corneal surface					
HOA RMS (μm)	3.02 (0.56)	3.28 (0.55 to 7.28)	4.14 (2.65)	2.38 (1.06 to 10.77)	0.380
Coma RMS (μm)	2.47 (0.53)	2.66 (0.11 to 5.58)	2.94 (1.68)	2.08 (0.50 to 5.82)	0.653
SA (μm)	− 0.21 (0.23)	− 0.17 (− 2.01 to 1.41)	− 0.30 (0.82)	− 0.06 (− 2.99 to 0.51)	0.110
Residual RMS (μm)	1.50 (0.30)	1.32 (0.53 to 4.22)	2.51 (2.22)	1.10 (0.67 to 9.70)	0.779
Posterior corneal surface					
HOA RMS (μm)	0.76 (0.16)	0.7 (0.13 to 1.99)	0.89 (0.43)	0.84 (0.21 to 1.73)	0.765
Coma RMS (μm)	0.60 (0.13)	0.59 (0.06 to 1.54)	0.68 (0.33)	0.70 (0.14 to 1.25)	0.806
SA (μm)	0.08 (0.08)	0.04 (− 0.46 to 0.63)	0.01 (0.22)	− 0.06 (− 0.21 to 0.77)	0.634
Residual RMS (μm)	0.38 (0.10)	0.33 (0.11 to 1.31)	0.48 (0.29)	0.36 (0.11 to 1.21)	0.399

*SD* = standard deviation; *RMS* = root mean square; *HOA* = high-order aberrations; *SA* = spherical aberration

### Ocular aberrometric changes

A significant reduction of ocular HOA RMS (*P* = 0.028) and primary coma RMS (*P* = 0.018) was found with the SCL. Specifically, mean HOA RMS changed from a pre-fitting value of 1.84 µm (SD: 1.40 µm; median: 1.24 µm; range: 0.36 to 5.04 µm) to a mean value with the CL of 0.73 µm (SD: 0.25 µm; median: 0.72 µm; range: 0.08 to 1.81 µm), whereas mean primary coma RMS changed from a pre-fitting value of 1.57 µm (SD: 1.33 µm; median: 0.99 µm; range: 0.23 to 4.81 µm) to a mean value with the CL of 0.45 µm (SD: 0.15 µm; median: 0.49 µm; range: 0.03 to 0.82 µm). Median HOA RMS was reduced by 41.9% and median primary coma RMS was reduced by 50.5%. There was a significant correlation between the change in VA with the CL and the change in primary coma RMS (r = 0.775, *P* = 0.041).

### Correlation of visual changes with pre-fitting variables

Statistically significant correlations of the change in VA achieved with the CL and a wide variety of pre-fitting variables were found: CDVA (r =  − 0.774, *P* < 0.001), ASTa (r =  − 0.449, *P* = 0.016), KMp (r = 0.417, *P* = 0.027), ASTp (r =  − 0.477, *P* = 0.010), MCT (r = 0.473, *P* = 0.011), HOA (r =  − 0.545, *P* = 0.003), primary coma (r =  − 0.425, *P* = 0.024) and residual RMS (r =  − 0.550, *P* = 0.002) of the anterior corneal surface, HOA (r =  − 0.470, *P* = 0.012), primary coma (r =  − 0.460, *P* = 0.014) and residual RMS (r =  − 0.497, *P* = 0.007) of the posterior corneal surface, and the power of the CL (r = 0.427, *P* = 0.021).

### Multiple linear regression analysis

A statistically significant linear relationship of the visual change in VA (LVA) with CL with different pre-fitting variables was obtained according to the following expression (*P* < 0.001, R^2^ = 0.878, adjusted R^2^ = 0.862, Durbin-Watson: 1.856):

$${\text{LVA}}\left( {{\text{logMAR}}} \right) = - {1}.{1}0{4} - 0.{766} \times {\text{CDVA}}\left( {{\text{logMAR}}} \right) + 0.0{18} \times {\text{CLP}} + 0.000{281} \times {\text{SHCL}}$$

where CDVA is the pre-fitting spectacle corrected distance visual acuity, CLP is the contact lens power of the SCL and SHCL is the sagittal lens of the SCL.

The normality of the unstandardized residuals distribution (*P* = 0.187) and the absence of influential points or outliers (mean Cook’s distance = 0.036 ± 0.057) confirmed the homoscedasticity of this model. Likewise, no multicollinearity was detected in the model (variance inflation factor between 1.277 and 2.650).

## Discussion

### Visual and aberrometric changes

In this study, changes occurring with the fitting of a specific model of SCL in corneas with KC were observed over a 3-month period. Factors correlating with visual improvement and a model to predict any improvement were investigated. A statistically significant improvement in VA has been observed in our sample of KC eyes, which is consistent with the results of previous clinical studies [6, 16, 22, 26, 27]. This good efficacy in terms of visual rehabilitation with the SCL was consistent with the efficacy in terms of optical correction, with mean ORx close to 0.00 D during the entire follow-up. Suarez et al. [6]. evaluated the efficacy and safety of the same SCL used in the current study, reporting a mean SLVA of 0.16 ± 0.25 logMAR and an improvement in VA of 2 or more lines in 56% of the eyes. This finding is consistent with those obtained in the current series in which mean SLVA was 0.10 ± 0.04 logMAR and 44.76% of the eyes reached an improvement in VA of 2 or more lines. This percentage was lower than that reported in the study by Suarez et al. [6] This can be explained by the fact that these authors started from a worse mean baseline VA (0.44 ± 0.45 logMAR), which is, according to our analysis, an advantageous situation for VA improvement. It should be remembered that a negative correlation between VA change and pre-fitting CDVA was found, which implies that subjects with worse pre-fitting CDVA experienced more improvement in VA with the SCL evaluated. Regarding VA changes between 1 and 3 months of CL wear, they were small in magnitude and not statistically significant, suggesting a stability of the VA during this period. This stability should be also confirmed in the long term.

The improvement in VA observed in our sample was associated with a significant change in ocular HOAs. Specifically, a significant decrease was observed with the CL in HOA and primary coma RMS, confirming the ability of this type of CL to minimize the aberrations that are present in KC eyes and to improve the ocular optical quality. However, the residual aberrations through SCLs were not always within the normative data for age and pupil size, which would have been the ideal situation [28, 29]. This was not always possible due to the presence of a not fully optimized meniscus in all cases in spite of adjusting most SCL parameters (it should be considered that no quadrant-specific designs were used), and even some patients could have some level of amblyopia associated. Furthermore, anterior corneal aberrations can be neutralized with the meniscus, but not always aberrations arising from the posterior corneal surface. Indeed, mean VA with the SCL was 0.10 ± 0.04 logMAR at the end of the follow-up and not all eyes reached the standard “20/20 or better VA”. This is consistent with previous series reporting that a great portion of KC patients fitted with SCLs do not reach normative levels for aberrations [30]. Additionally, a significant correlation was found between the decrease in comatic aberration and the improvement in VA (r =  − 0.775, *P* = 0.041). In this way, eyes with higher amounts of coma improved more after SCL fitting than eyes with lower levels of coma. In our series, there were 31% of eyes that did not improve VA after the SCL fitting and this was largely due to the presence of low amounts of coma aberrations as the KC was central or was incipient. This should be considered when fitting SCLs, as those eyes with less visual loss and lower levels of visual quality are expected to show minimal or limited improvements. Montalt et al. [27] also showed a decrease in ocular coma and HOAs with the use of a SCL, with a 55% decrease of total HOAs. These authors commented that despite this reduction, the aberration values with the SCL in KC were still higher than in normal corneas, suggesting that despite masking the irregularities of the corneal surface, aberrations on the posterior surface of the cornea and internal aberrations are not compensated [27]. Alipour et al. [18] also observed a statistically significant reduction in coma and trefoil aberrations with the use of SCLs in eyes with KC implanted with intracorneal ring segments.

Concerning the correlations of pre-fitting variables with the VA change achieved with the SCL evaluated, they were statistically significant for spectacle CDVA, anterior and posterior corneal astigmatism, posterior keratometry, MCT, anterior and posterior HOAs and the power of the CL. This confirms that the VA change achievable with the SCL evaluated in KC corneas can be predicted using all these variables, although the level of accuracy is not similar for all of them. The strongest correlation was found between the VA change and the pre-fitting CDVA value and it was negative. This means that patients with the worst initial VA were those who presented the greatest visual improvement with the fitting of the SCL. Considering that those eyes with worse VA are normally KC eyes with more aberrated corneas, it is normal that better VA would be expected as more ocular optical quality can be induced [16]. Indeed, the rest of significant correlations detected revealed that more VA improvement could be achieved in those eyes with more significant signs associated to moderate to severe stages of KC, such as higher levels of anterior and posterior corneal astigmatism, higher amounts of HOAs, greater curvature of the posterior corneal surface and lower MCT [31, 32].

### Post-lens meniscus characterization

The characterization of the post-lens meniscus by OCT showed that the central, nasal, and temporal vaults decreased significantly after 1 month of SCL wear. This is the result of the progressive indentation of the periphery of SCL into the conjunctival tissue [33]. Vincent et al. [34] found that the central vault of the ICD16.50 SCL significantly decreased an average of 76 ± 8 μm after 8 h of use. Furthermore, they observed that 50% of the reduction occurred in the first 45 min, 75% in 2 h and after 4 h of use the decrease was not statistically significant [34]. Likewise, these authors found a statistically significant but not clinically relevant change associated in ORx [34]. Similarly, a reduction of the vault has been reported with other SCL designs. Bray et al. showed a decrease in the vault of 83 ± 22 μm after 6–8 h of CL use, with statistically significant changes in ORx associated [35]. Rathi et al. [36] observed a decrease in the central vault in 90% of their sample after 4 h of use of a SCL, ranging from an initial value of 680 ± 421 μm to 589 ± 355 μm. Courey and Michaud [37] showed a decrease in the central vault of 70 ± 9.8 μm after 6 h of use of a specific model of SCL. Otchere et al. [38] evaluated the decrease in the central vault of 3 different types of SCLs, obtaining a mean value of 34 ± 48 μm after 1 h of use. Furthermore, these authors observed that the vault loss depended on the initial magnitude of the vault. These mean decreases reported in previous studies were close to those obtained in our sample after 1 month of CL wear. Furthermore, previous studies indicate that most changes in SCL vault occurred in the initial period of CL wear, no significant changes were found in our sample in the characterization of horizontal post-lens meniscus between the first and third months of CL wear.

A significant difference between the nasal and temporal vault was found in our sample during the initial post-fitting period which could be due to a temporal decentration of the SCL, a greater indentation in the nasal area or a decentered CL position as a consequence of the asymmetry of the corneal surface in KC [39]. Courey and Michaud [37] showed a smaller vault on the nasal side than on the temporal that was attributed by the authors to the toric nature of the sclera. Concerning the difference in nasal-temporal vault in our study over time, it was not statistically significant after 3 months of CL use, which could indicate that the SCL stabilizes over time. This may be related to some level of conjunctival molding with the use of the SCL over time, as has been demonstrated using Fourier-domain profilometry to characterize the corneo-scleral profile [33].

Considering the distribution of the sagittal heights of the SCL fitted that were needed in the sample and that most eyes had a KC grade I and II, it can be concluded that a trial lens with a sagittal height between 4000 and 4400 µm is needed. This can be used for fitting recommendations when specific instruments for measuring the ocular sagittal heights are unavailable. According to this, sagittal heights of 4600 µm or higher would be needed for the trial lens when fitting the SCL evaluated in advanced KC.

### Corneal tomographic changes

Concerning the evaluation of corneal tomographic changes, these did not reach statistical significance. This finding is in contrary to other studies that have reported significant changes in corneal shape and thickness in the short term [23, 24]. Serramito et al. [24] showed that there was a statistically significant corneal thinning in the inferior region of KC eyes fitted also with the ICD16.50 SCL and in the superior region of KC eyes implanted with intracorneal ring segments. A downward trend in MCT and CCT was observed in our series, but both did not reach statistical significance. Vincent et al. [40] reported a small and statistically significant amount of edema after 8 h of use of the ICD16.50 SCL in healthy adults. Specifically, a mean increase of 10.23 ± 5.77 μm in corneal thickness was observed which corresponded to 2% edema [40]. In another study, these same authors investigated the variation of edema over time, observing that the corneal thickness swelled after 15 min of CL use, stabilizing 45 min after CL insertion, reaching its maximum point after 90 min with a 1.18 ± 0.20% of edema, and gradually thinning after 2 h of use [41]. Possibly, in our sample, changes in corneal thickness associated to the initial use of the lens had been already stabilized when they were evaluated after 1 and 3 months of CL use. Similarly, minimal and no significant changes in anterior and posterior corneal shape and aberrations (6-mm pupil) were found, confirming the stability of the cornea with the use of the SCL during the first three months. Serramito et al. [23, 24] only found significant changes in anterior and posterior spherical aberrations after 8 h of SCL use, although more HOAs changed significantly when intracorneal ring segments had been implanted. In any case, a high variability was observed in corneal tomographic changes in the sample evaluated in the current study, confirming that the impact of the SCL on the cornea may vary between KC individuals.

### Prediction model of visual change with the SCL

Finally, a multiple linear regression equation was obtained to predict the final VA through a series of initial data. This equation proposes the prediction of the VA change with the SCL evaluated from three variables (pre-fitting spectacle CDVA, lens power, and lens sagittal height) that can be obtained in the pre-fitting and first trial visits. Therefore, more VA improvement can be expected when fitting this SCL in those eyes with worse pre-fitting VA and fitted with SCL with lower sagittal heights and optical power. According to this, the ideal candidate for optimizing the visual quality with this type of SCL would be an advanced KC eye requiring a moderate to high myopic correction using a moderate to low sagittal height of the lens. The combination of the aberrometric profile of a negative powered SCL with a low sagittal height potentially associated to a low central vault (lower optical contribution of the post-lens meniscus as it would be thinner) may be associated to a potentially better optical quality and consequently may explain the contribution of these two factors to the predicting equation. This should be investigated in future investigations analyzing the optical profile of these SCLs as well as the optical impact of the post-lens meniscus. This prediction model has been verified and fulfilled the requirements to ensure its validity. The equation was able to predict the VA of the patients in the sample with very good results where only 10.3% of cases in which the error would be greater than 0.1 logMAR.

### Limitations

Our study had several limitations. First, the study conducted was retrospective in nature. Second, a larger sample size especially for the development of predicting models would be more useful. Therefore, the current version of this model should only be considered preliminary and could be refined including data from larger samples. Furthermore, the measurements of the meniscus with the OCT were done by a single person manually so biases may have been present.

## Conclusions

In conclusion, the ICD16.50 SCL provides an efficacious visual rehabilitation in KC due to the improvement of VA and the correction of the low and high-order ocular aberrations. The visual result achievable with this SCL is predictable from a linear equation relating pre-fitting spectacle CDVA, power and sagittal height of the SCL fitted.

## Data Availability

Not applicable.
